# Exploring the exoproteome of the parasitic nematode *Anisakis simplex* (s. s.) and its impact on the human host – an *in vitro* cross-talk proteomic approach

**DOI:** 10.3389/fimmu.2025.1509984

**Published:** 2025-02-03

**Authors:** Robert Stryiński, Ewa Fiedorowicz, Jesús Mateos, Aneta Andronowska, Elżbieta Łopieńska-Biernat, Mónica Carrera

**Affiliations:** ^1^ Department of Biochemistry, Faculty of Biology and Biotechnology, University of Warmia and Mazury in Olsztyn, Olsztyn, Poland; ^2^ Department of Food Technology, Institute of Marine Research, Spanish National Research Council, Vigo, Spain; ^3^ Clinical Pharmacology Group, Health Research Institute of Santiago de Compostela (FIDIS), Santiago de Compostela, Spain; ^4^ Department of Hormonal Action Mechanisms, Institute of Animal Reproduction and Food Research, Polish Academy of Sciences, Olsztyn, Poland

**Keywords:** *Anisakis simplex*, Caco-2 cell line, extracellular vesicles, parasite-host interactions, proteomics

## Abstract

**Introduction:**

Anisakis simplex sensu stricto (s. s.) is one of the most widespread parasitic nematodes of marine organisms, with humans as accidental hosts. While many studies have explored nematode biology and host interactions, the role of extracellular vesicles (EVs) as signaling molecules in parasitic nematodes is less understood.

**Materials and methods:**

Therefore, the proteins present in the EVs of A. simplex (s. s.) (Anis-EVs) were identified. In addition, a cross-talk proteomic approach was used to identify differentially regulated proteins (DRPs) in the proteome of the human intestinal epithelial cell line (Caco-2) co-cultured with L3 larvae of A. simplex (s. s.) or directly exposed to two concentrations (low or high) of Anis-EVs. In addition, DRPs were identified in the proteome of A. simplex (s. s.) larvae affected by co-culture with Caco-2. To achieve this goal, the shotgun proteomics method based on isobaric mass labeling (via tandem mass tags; TMT) was used with a combination of nano high-performance liquid chromatography (nLC) coupled with an LTQ-Orbitrap Elite mass spectrometer. In addition, ELISA assays were used to demonstrate if Caco-2 respond to A. simplex (s. s.) larvae and Anis-EVs with significant changes in selected cytokines secretion.

**Results:**

The results of this study indicate the anti-inflammatory character of Anis-EVs in relation to Caco-2. At the same time, direct treatment with Anis-EVs resulted in more significant changes in the Caco-2 proteome than co-culture with L3 larvae.

**Discussion:**

The results obtained should lead to a better understanding of the molecular mechanisms underlying the development of A. simplex (s. s.) infection in humans and will complement the existing knowledge on the role of EVs in host-parasite communication.

## 1 Introduction

*Anisakis simplex* sensu stricto (s. s.), a widespread and significant parasitic nematode of marine organisms, is characterized by a complex life cycle. Many fish, cephalopod species and marine mammals have been identified as hosts of *A. simplex* (s. s.) (1). Humans may become accidentally infected with *A. simplex* (s. s.) through consumption of parasitized row or under-cooked fish and fishery products containing larvae. This may pose a serious health risk because the parasites are able to penetrate mucous membranes of the gastrointestinal tract and cause damage to the gastric and intestinal walls (2). The symptoms of *A. simplex* (s. s.) infection relate to the gastrointestinal tract and include epigastralgia, nausea, and diarrhea (3). Some patients develop allergy to parasite antigens. The *Anisakis*-associated allergic response is characterized mainly by production of specific IgE, tissue eosinophilia, angioedema, urticaria, and it may lead to life threatening anaphylactic shock (4–6). Therefore, *A. simplex* (s. s.) was acknowledged as biohazardous organism (7). The pathological changes caused by nematodes of the *Anisakis* genus are known as anisakiasis (8). The presence of *Anisakis* larvae in the intestinal wall induces a Th2-type immune response characterized by an increased host production of eosinophils and elevated secretion of cytokines (IL-4, -5, -10, -13, -25, -33), the symptoms like those of food allergy (9–11). Interestingly, the exposure to *Anisakis* larvae was reported as a potential risk factor for gastric or colon adenocarcinoma (12). In addition, recent studies have shown that *A. simplex* (s. s.) body extracts induced an inflammation (13). Moreover, compounds excreted/secreted by *A. simplex* (sensu lato) into host environment may have an inhibitory effect on lymphocyte blastogenesis (14). Thus, these nematode secretions may have immunomodulatory effects interfering with host immune responses.

Anisakiasis is becoming a global is becoming a global problem in an era of widespread travel and rapid growth of international trade. The growing popularity of exotic dishes made from raw fish and cephalopods, and the general practice of undercooking seafood also contribute to the spread of the disease (15, 16).

The finding that nematodes release extracellular vesicles (EVs) which can enter host cells, has been a breakthrough discovery in parasite research (17, 18). EVs are small membrane-bound vesicles formed in the cell and released via exocytosis (19). Various proteins, lipids, and nucleic acids capsulated into EVs may affect functions of neighboring cells. It has been demonstrated that EVs not only contribute to importantly biological functions such as tissue repair, neural communication, and the transfer of pathogenic proteins (20) but also regulate cellular functions, including motility and polarization, immune responses, and development, and contribute to cancer and neurodegeneration (21). The parasitic EVs were demonstrated for the first time in *Trypanosoma brucei* (17). In recent years, several protozoa and nematodes were also found to secrete EVs into the organisms of living hosts (18). In parasitic nematodes, EVs were reported to have an important role in establishing and maintaining the infection. There is a vast body of evidence that EVs can act as signal molecules in parasite-parasite and parasite-host communication, leading to the infection of the host (22–25). Therefore, it cannot be excluded that EVs from *A. simplex* (s. s.) may play an important role in the regulation of host immune defenses.

Although studies of the parasite-host interactions led to the identification of potential targets for diagnosis and therapy of nematodiases (26, 27), further studies are required to search for new and more specific targets to treat these diseases more effectively (18, 22). In this context, evidence that EVs can act as signal molecules in parasite-host interactions may pave the way to novel strategies for nematode infection control. So far, several approaches have been employed to study the biology of parasitic nematodes and their interactions with hosts. Recently, when researching the complicated dynamics of host-parasite interactions, researchers are increasingly relying on omic approaches, in particular cross-talk proteomic analyses, to decipher the molecular relationships. By simultaneously profiling all proteins expressed by both the parasite and its host, cross-talk proteomic studies offer unprecedented insights into the complex interplay between organisms within a specific ecosystem. This comprehensive approach enables the identification of key effectors of the parasite and host factors involved in the modulation of immune responses, tissue remodeling and other physiological processes. In addition, cross-talk proteomic analyses using shotgun proteomics approach facilitate the discovery of new biomarkers for disease diagnosis and prognosis as well as the identification of potential targets for therapeutic intervention (28–31).

Therefore, the proposed study aimed to investigate the proteomic profile of the host-parasite relationship using the human intestinal epithelial cell line (Caco-2) co-cultured with whole *A. simplex* (s. s.) L3 larvae or directly treated with EVs produced by *A. simplex* (s. s.) L3 larvae (Anis-EVs) to evaluate the potential role of the parasite’s EVs in the host response to infection.

## 2 Materials and methods

### 2.1 *In vitro* culture of *A. simplex* larvae

The study was performed on the L3 larval stage of *Anisakis simplex* (s. s.) (Rudolphi, 1809) Dujardin, 1845. Nematodes were isolated from Baltic herrings (*Clupea harengus* Linnaeus, 1758) caught in waters of local major fishing area FAO 27.3.d (Baltic Sea). The specific culture conditions for EVs isolation were adapted to the requirements developed by specialists investigating the biology of EVs and *in vitro* culture was conducted according to accepted guidelines for parasitic helminths (32, 33). The L3 larvae of *A. simplex* (s. s.) were cultured for 3 days in RPMI-1640 serum-free medium (37°C, 5% CO_2_) in 6-well plates, to collect the largest possible number of EVs needed for all analyses and subsequent cultures (34). Medium was collected at the end of the culture and used for EVs isolation. After the culture, the larvae were taxonomically identified using conventional Real-time PCR to amplify the ITS region with use of Anis Sensitive Sniper RT-PCR Kit (A&A Biotechnology, Gdynia, Poland) as described before (35). All the larvae were identified as *Anisakis simplex* (s. s.).

### 2.2 Extracellular vesicles derived from *Anisakis simplex*

#### 2.2.1 EVs isolation

The procedure for the enrichment of EVs from *A. simplex* (s. s.) (Anis-EVs) was conducted according to special considerations for studies of extracellular vesicles from parasitic helminths by ultracentrifugation method (32, 33). In brief, the first step of centrifugation was performed at 300 × g for 10 min in 15 mL falcon tubes with the use of Centrifuge 5804R (Eppendorf, Warsaw, Poland) to pellet the debris. Then, the samples were transferred to new 13.2 mL ultra-clear tubes (Beckman Coulter, Poland, cat. no C14277) and subjected to differential ultracentrifugation to remove apoptotic bodies, and medium/large EVs. At each of these steps (2,000 × g for 10 min and 10,000 × g for 30 min) the pellet was thrown away, and the supernatant was used for the following step. Then, the supernatant was passed through a 0.22 μm PVDF filter (Merck, MA, USA, cat. no GVWP04700). The filtered medium supernatant was subjected to the ultracentrifugation at 100,000 × g for 70 min with Optima L-100 XP Ultracentrifuge (Beckman Coulter, Brea, CA, USA) and SW 41 Ti Rotor (Beckman Coulter, Brea, CA, USA). At the end, the pellet containing Anis-EVs was washed in a 1 mL of Dulbecco′s Phosphate Buffered Saline (DPBS; Merck, Poland, cat. no 59331C) to eliminate any other contaminations, and centrifuged last time at the same high speed of 100,000 × g for 70 min. The pellet containing Anis-EVs was resuspended with 100 µL of DPBS and stored at - 80°C until the time of next step.

#### 2.2.2 Nanoparticle tracking analysis

The size distribution and number of Anis-EVs were analyzed by measuring the rate of Brownian motion of each particle using a NanoSight NS300 instrument (Malvern Instruments, Ltd., Wiltshire, UK). The Anis-EVs samples were diluted 20 - 100 times with DPBS (Merck, MA, USA, cat. no 59331C) until the number of particles in the field of view was less than 1000. The purity of DPBS was checked on NTA and the absence of any particles that could affect the measurements of the Anis-EVs samples was confirmed. Samples were analyzed using NanoSight NS300 NTA software (version 2.3, Malvern Instruments, Ltd., Wiltshire, UK).

#### 2.2.3 Visualization by transmission electron microscopy

The morphology of Anis-EVs was evaluated by TEM as described previously (36). In brief, the droplets of purified EVs were transferred to copper formvar/carbon-coated grids with 200 mesh size (Agar Scientific, UK), adsorbed for 20 min, washed in sterile distilled water, and contrasted with 2.5% aqueous uranyl acetate (Agar Scientific, UK) for 2 min. Images were acquired using a JEM 1400 TEM (JEOL Ltd. Tokyo, Japan, with Morada TEM CCD camera, Olympus, Hamburg, Germany) at 80 kV.

#### 2.2.4 Purity of isolated EVs

The purity of the isolated Anis-EVs was calculated according to Webber and Clayton (37) using the ratio of particles to protein. The method clearly discriminates pure vesicle preparations from those replete with contaminating protein, proposing a ratio of 3×10^10^ particles per µg of protein, or greater as high purity. Total protein concentration in the supernatants was measured by bicinchoninic acid method (Pierce BCA Protein Assay Kit, Thermo Fisher Scientific, Waltham, MA, USA) and the number of Anis-EVs was analyzed by NTA as described above.

### 2.3 *In vitro* culture of host cells

**Figure 1:**
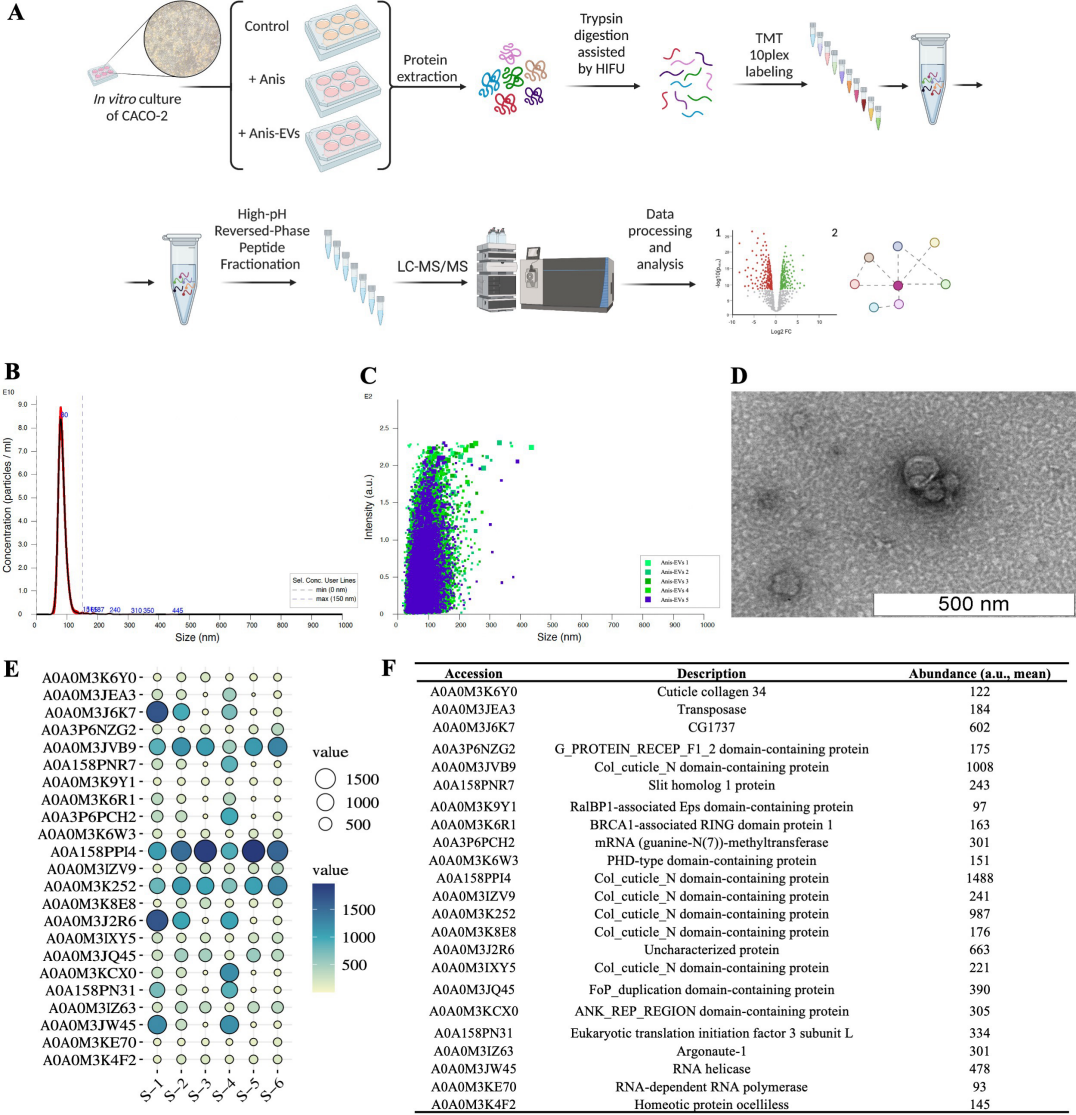
Workflow and results of the characterization of extracellular vesicles released by *Anisakis simplex* (s. s.). **(A)** Workflow of the *in vitro* cultures and proteomic analysis. **(B, C)** Concentration and intensity of Anis-EVs measured by Nanoparticle Tracking Analysis. The mean size of Anis-EVs varies around 85.3 nm (2.22 × 10^12^ particles/mL; 98.7% of all particles/ml; SD = 19.9 nm) **(D)** Representative transmission electron micrographs of Anis-EVs. The scale in the figure indicates 500 nm. **(E)** The balloon plot of proteins identified between the EVs samples (n = 6) isolated from post-culture medium of *A. simplex* (s. s.). Balloon size and colors were used to indicate the abundance ratio values (Log2) for each biological replicate. **(F)** The detailed list of proteins identified in Anis-EVs with accession number to UniProt Database and mean abundance [a.u.] between samples.

The human intestine epithelial cell line, Caco-2, was acquired from the biobank of Department of Biochemistry, Faculty of Biology and Biotechnology, University of Warmia and Mazury in Olsztyn, Poland. Cells at passage 53-56 were used in the study. The Caco-2 cells were cultured in the filtrated DMEM medium (Dulbecco’s modified Eagle’s medium, Sigma-Aldrich, St. Louis, MO, USA, cat. no. D6429) containing 10% of heat-inactivated FBS (Gibco, Thermo Fisher Scientific, Waltham, MA, USA, cat. no. 16000044), 1% nonessential amino acids serum (Gibco, Thermo Fisher Scientific, Waltham, MA, USA, cat. no. 11140050), and 1% of penicillin-streptomycin solution (Sigma-Aldrich, St. Louis, MO, USA, cat. no. P4333) (38). The cells were seeded at a density of 2.5×10^5^ cells/well in 6-well plates and the culture medium was changed every 2 days (37°C, 5% CO_2_ and humidity of 95%). Studies were performed on 2-3 days post confluent Caco-2 cells, which were divided into two groups, one for the culture with whole *A. simplex* (s. s.) larvae, and second for the direct culture with Anis-EVs (**Figure 1A**).

#### 2.3.1 Caco-2 with *Anisakis* larvae

When Caco-2 cells were 2-3 days post confluent, the L3 larvae of *A. simplex* (s. s.) were added to each well in number of 5 per well (control without the larvae). The co-culture was conducted for 48 h. At the end of the culture, the host cells, as well as *A. simplex* (s. s.) larvae were collected for protein extraction. The culture medium was used to determine the concentration of selected cytokines (see 2.10.).

#### 2.3.2 Caco-2 with Anis-EVs

When Caco-2 cells were 2-3 days post confluent, the Anis-EVs were added to the medium in two concentrations: low (Anis-EVs Low) and high (Anis-EVs High) (1 × 10^9^ and 1 × 10^10^ Anis-EVs per well, respectively, resulting in around 500 or 5,000 Anis-EVs per cell, respectively; control without Anis-EVs). The Caco-2 cells with/without Anis-EVs were cultured for 48 h. At the end of the culture, the host cells were collected for protein extraction and medium was used to determine the concentration of selected cytokines (see 2.10.).

### 2.4 Protein extraction

Total protein was extracted from the collected samples i.e., from the host cells cultured with (treatment, n = 6) and without (control, n = 4) L3 larvae of *A. simplex*, and from host cells cultured with (treatment; two concentrations of Anis-EVs, 2 × n = 3) and without Anis-EVs (control, n = 3). The protein was also extracted from L3 larvae of *A. simplex* (s. s.) after the co-culture with host cells (treatment, n = 6; control samples were larvae cultured without cells, n = 4). The protein was also extracted from Anis-EVs (n = 6).

To extract total protein, the samples were incubated in 0.2 mL of lysis buffer on ice (own modification of RIPA buffer; 50 mM Tris-HCl pH 8, 150 mM NaCl, 0.1% Triton X-100, 0.5% sodium deoxycholate, 0.1% SDS). After the incubation a sonication was conducted (3 cycles, 30 s pulses; IKA-Werke, Staufen, Germany). Then, the protein extracts were centrifuged (16,000 × g, 30 min, 4°C). Total protein concentration in the supernatants was measured by bicinchoninic acid method (Pierce BCA Protein Assay Kit, Thermo Fisher Scientific, Waltham, MA, USA). A total of 100 μg of the protein from host cell samples (19 samples in total), and *A. simplex* (s. s.) larvae samples (10 samples in total), and a total of 20 μg of the protein from Anis-EVs samples (6 samples in total) was transferred into new tubes and overnight acetone precipitation was performed (1.8 mL of cold acetone was added to each sample and incubated overnight at - 20°C). Then, samples were centrifuged (15,000 × g, 10 min, 4°C), and tryptic digestion with the simultaneous application of high-intensity focused ultrasounds (HIFU) was carried out, as described previously by Stryiński et al. (39, 40).

### 2.5 TMT labeling and reversed-phase fractionation

The TMT 10-plex isobaric label reagents (0.8 mg, Thermo Fisher Scientific, Waltham, MA, USA) were resuspended in 41 μL of anhydrous acetonitrile and added to 25 or 100 μg of protein digest, as described by Stryiński et al. (40). Within the experiment, samples were labeled with TMT10-plex as indicated in **Supplementary Table 1**. Samples within each TMT were combined in a new tube at equal amounts according to the manufacturer’s instructions. To increase the number of peptide identifications, eliminate the interference from co-isolated ions and achieve results comparable to the MS^3^-based methods (41, 42), the combined sample was fractionated using a Pierce High-pH Reversed-Phase Peptide Fractionation Kit (Thermo Fisher Scientific, Waltham, MA, USA) following the manufacturer’s instructions. The peptide concentration in each fraction was determined by colorimetric analysis using the Quantitative Colorimetric Peptide Assay (Thermo Fisher Scientific, Waltham, MA, USA) following the manufacturer’s instructions. Then, fractions were evaporated to dryness using vacuum centrifugation (SpeedVac concentrator, Thermo Fisher Scientific, Waltham, MA, USA). The samples were stored at -80°C until further analysis.

### 2.6 LC-MS/MS analysis and data processing

Peptide fractions (eight fractions from each TMT labeling; 32 samples in total) were acidified with 0.1% formic acid and analyzed by nLC-MS/MS using a Proxeon EASY-nLC II liquid chromatography system (Thermo Fisher Scientific, Waltham, MA, USA) coupled to an LTQ-Orbitrap Elite mass spectrometer (Thermo Fisher Scientific, Waltham, MA, USA) as described previously (42, 43). All acquired MS/MS spectra were analyzed using SEQUEST-HT (Proteome Discoverer 2.4 package, Thermo Fisher Scientific, Waltham, MA, USA) against a reference proteome of *Homo sapiens* available in the UniProt/TrEMBL database (proteome ID: UP000005640; # of entries 82,492 entries) or against a reference proteome of *Anisakis simplex* (proteome ID: UP000267096; # of entries 20,779 entries). The following restrictions were used: full tryptic cleavage with up to 2 missed cleavage sites and tolerances of 10 ppm for parent ions and 0.06 Da for MS/MS fragment ions. TMT-labeling (+229.163 Da on N-termini and lysine residues) and carbamidomethylation of cysteine (+57.021 Da) were set as fixed modifications. The permissible variable modifications were methionine oxidation (+15.994 Da), acetylation (+42.011 Da) of the N-terminus of the protein, and deamidation (+0.984 Da) of asparagine and glutamine. Moreover, searching parameters included four maximal dynamic modification sites.

### 2.7 Statistical analysis of proteomics data

The results were subjected to statistical analysis to determine the peptide false discovery rate (FDR) using a decoy database and the Target Decoy PSM Validator algorithm (44). The FDR was kept below 1%, and for further analysis, only proteins meeting selected criteria were submitted: a) master proteins, b) proteins quantified with at least two unique peptides, and c) proteins with different protein IDs. Relative quantification was performed using the Quantification Mode and normalization was conducted against total peptide amount (Proteome Discoverer 2.4 package, Thermo Fisher Scientific, Waltham, MA, USA). After relative quantification, several filters were applied to obtain the final list of DRPs: a) at least a 1- or 1.5-fold change in normalized ratios and b) ANOVA and Tukey HSD *post-hoc* test (*p*-value < 0.05). Volcano-plot representations of identified DRPs were plotted. Furthermore, the distribution of common and unique DRPs identified in the three different cultures of Caco-2 cells (co-culture with *A. simplex* (s. s.) larvae, and treatment with two concentrations of Anis-EVs) was analyzed and visualized using the Venn Diagrams tool (https://bioinformatics.psb.ugent.be/webtools/Venn/).

### 2.8 Functional categories of identified proteins

The final list of DRPs obtained in each experiment was classified into three different Gene Ontology categories (GO; biological processes, cellular components, and molecular functions) and assigned to the metabolic pathways in which they are involved. Analyses were performed with clusterProfiler (45) and Pathview (46), two R packages established for GO and pathway enrichment analysis (*p*-value < 0.05).

### 2.9 Protein-protein interactions network analysis

Network analysis was performed by submitting the DRPs dataset to Cytoscape (v. 3.8.0.; NIGMS, Bethesda, MD, USA), a software platform for visualizing complex networks, and analyzed by stringApp (v. 1.5.1.) (47). Full STRING networks (the edges indicate both functional and physical protein associations) have been identified by comparing the input data with the background of the *Homo sapiens* or *Anisakis simplex.* All interactions were indicated in the context of co-expression, co-occurrence and based on the appearance of information on the interactions between these proteins in different databases. The remaining input proteins that are not associated with any other protein were excluded from the network visualizations. The Markov Cluster Algorithm (MCL) was used for clustering the obtained networks (inflation parameter = 3).

### 2.10 Cytokines levels measurement

Levels of selected cytokines (IL-6, IL-8, IL-10) were examined using enzyme-linked immunosorbent assay (ELISA) kits obtained from Mabtech (IL-6, Nacka Strand, Sweden, cat. no. 3460-1H-20), Diaclone (IL-10, Besançon Cedex, France, cat. no. 851.540.001), and BD Biosciences (IL-8, San Jose, CA, USA, cat. no. 555244) according to the manufacturers’ protocols. Samples were run in triplicate. Results were calculated by comparison with a standard curve. Ordinary two-way ANOVA and Dunnett’s multiple comparisons tests were used to examine differences between quantitative values. Statistical significance was defined as *p*-value ≤ 0.05 (GraphPad Prism software version 8, San Diego, CA, USA).

## 3 Results

### 3.1 Characterization of *Anisakis*-derived EVs and their proteomic composition

The characteristics of EVs from *A. simplex* (s. s.) was performed according to accepted guidelines for parasitic helminths (32). NTA and TEM methods were used to characterize Anis-EVs and evaluate their size distribution and morphology, respectively. The NTA revealed a population of particles smaller than 150 nm. The mean size of Anis-EVs oscillates around 85.3 nm (2.22 × 10^12^ particles/mL; 98.7% of all particles/mL; SD = 19.9 nm) (**Figure 1B**). Particle individual intensity is shown in the **Figure 1C**. Protein quantification yielded 62.4 µg/mL ± 11.78, which at a concentration of 2.22 × 10^12^ particles/mL gives a purity ratio of 3.55 × 10^10^ P/µg and indicates that the EVs obtained are high purity preparations.

TEM revealed uniform particles smaller than 150 nm with identifiable lipid bilayer membranes, circular cross sections, and a characteristic “cup shape” (**Figure 1D**).

Proteomic analysis of Anis-EVs showed 23 annotated proteins enriched in isolated vesicles (**Figures 1E, F**). The results of the protein identification search are summarized in the **Supplementary Table 2**.

### 3.2 Co-culture of Caco-2 with Anisakis larvae or treatment with Anis-EVs led to changes in the proteome of host cells

**Figure 2:**
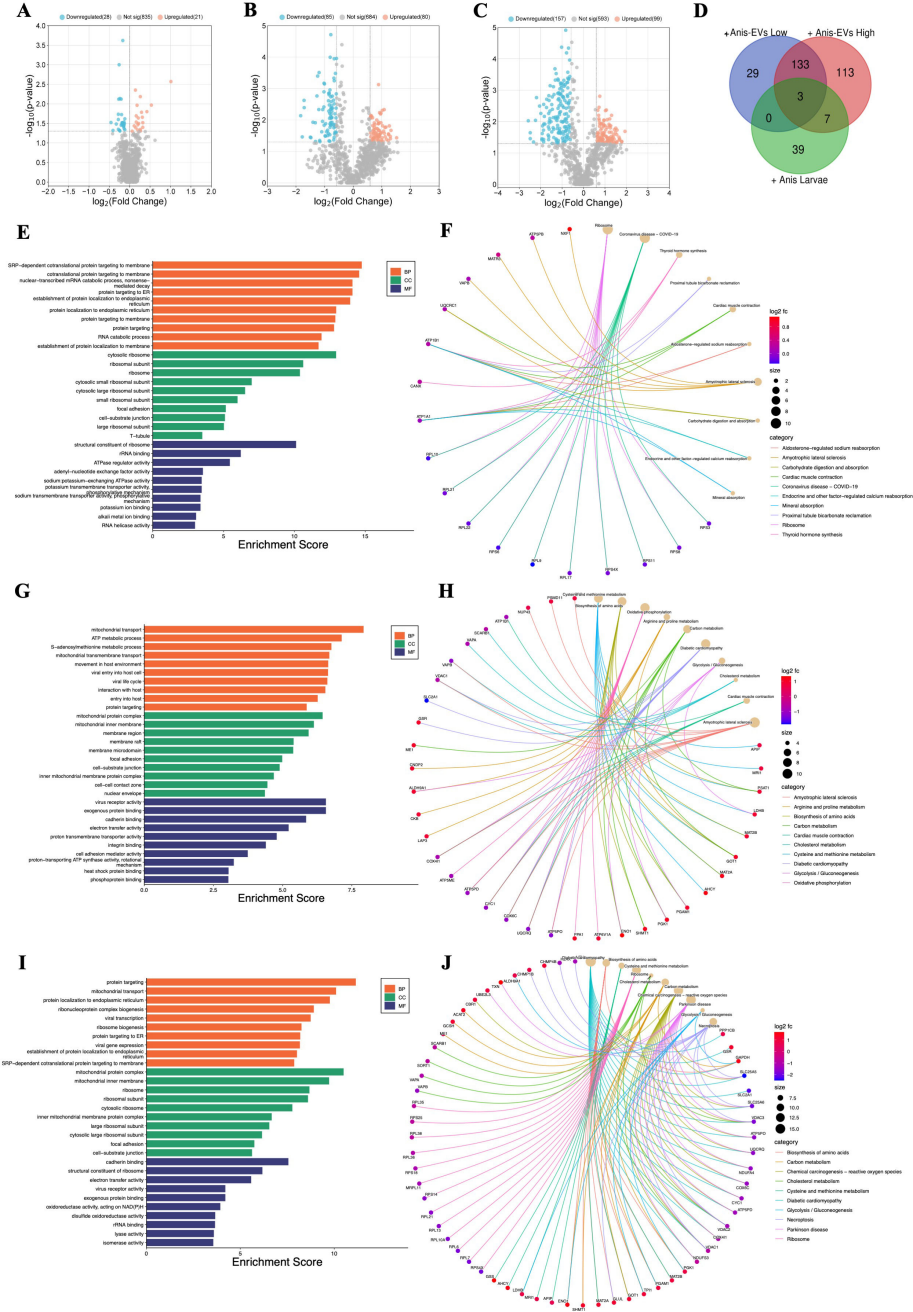
Functional characterization of the differentially regulated proteins (DRPs) identified in Caco-2 cells after different treatments. **(A)** Influence on Caco-2 cells cocultured with *A*. *simplex* (s. s.) larvae (FC = 1.0, *p*-value ≤ 0.05), **(B)** and of treatment with low, and **(C)** with high concentration of Anis-EVs (FC = 1.5, *p*-value ≤ 0.05). In presented volcano plots, the most upregulated proteins were towards the right (red), the most downregulated proteins were towards the left (blue), and out of them the most statistically significant proteins were towards the top. **(D)** The analysis of distribution of common and unique DRPs in three different cultures of Caco-2 cells: with low (+Anis-EVs Low) and high (+Anis-EVs High) concentration of Anis-EVs, and with *Anisakis* larvae (+Anis Larvae). **(E-J)** The visualizations of GO analysis (left) and pathways enrichment analysis results (right) obtained for DRPs identified in Caco-2 cells: **(E, F)** co-cultured with *A*. *simplex* (s. s.) larvae, and treated with **(G, H)** low and **(I, J)** high concentrations of Anis-EVs. Detailed results can be found in the **Supplementary Tables 7**-**12**.

The proteome of Caco-2 cells treated with Anis-EVs or co-cultured with *A. simplex* (s. s.) larvae was analyzed using LC-MS/MS. Statistical analysis of the data led to the creation of Volcano plots to represent DRPs (**Figures 2A–C**). The proteome of Caco-2 cells has been slightly altered when co-cultured with whole larvae of *A. simplex* (s. s.) (**Figure 2A**). Twenty-one proteins were found to be upregulated, and 28 proteins were downregulated compared to cells cultured without *Anisakis* larvae (FC = 1.0; *p*-value < 0.05; **Supplementary Table 3**).

Significant changes were observed in the proteome of Caco-2 cells when they were cultured directly with Anis-EVs (**Figures 2B, C**). The response of the cells to the Anis-EVs varied depending on the amount of EVs used. Proteomic analysis of cells cultured with a low dose of EVs (Anis-EVs Low; ~500 particles per cell; **Figure 2B**) showed that 165 proteins were differentially regulated compared to the control, with 80 proteins being upregulated and 85 proteins being downregulated (FC = 1.5; *p*-value < 0.05). On the other hand, analysis of cells cultured with a high dose of EVs (Anis-EVs High; ~5,000 particles per cell; **Figure 2C**) showed 256 DRPs, with 99 being upregulated and 157 being downregulated (FC = 1.5; *p*-value < 0.05). The specific proteins that were differentially regulated can be found in **Supplementary Tables 4** and **5**.

The analysis of distribution of common and unique DRPs in three different cultures of Caco-2 cells showed that the same three proteins (VAPB, CANX, ATP1B1) were affected by all three factors (larvae and two concentrations of EVs). Seven proteins were common to high-dose EVs (Anis-EVs High) treatment and co-culture with larvae (HNRNPA0, RPL21, DDX18, RPS4X, DDX21, IGF2BP1 ATP1A1). Additionally, there were 133 DRPs that were common to both groups treated with different concentrations of EVs (**Figure 2D**, **Supplementary Table 6**).

### 3.3 DRPs are associated with diverse biological functions and pathways in host cells

Proteins identified in Caco-2 cells after co-culture with *A. simplex* (s. s.) larvae were subjected to GO analysis. The results at the level of significance (*p*-value < 0.05) showed that this group is involved in 170 biological processes (BP), these proteins are located in 69 different cellular components (CC) and perform 49 different molecular functions (MF) (**Supplementary Table 7**). The most significant hits are presented in **Figure 2E**. These proteins were also subjected to metabolic pathway enrichment analysis, which identified 15 different metabolic pathways involving these proteins, including the *ribosome* (10 DRPs; hsa03010) or *carbohydrate digestion and absorption* (2 DRPs; hsa04973) (**Figure 2F**, **Supplementary Table 8**).

DRPs identified in Caco-2 cells after their treatment with Anis-EVs were also subjected to GO and pathways enrichment analysis. The low concentration of extracellular vesicles (Anis-EVs Low) influenced the differential regulation of 161 proteins, which resulted in 404 BPs, 90 CCs, and 72 MFs (**Figure 2G**, **Supplementary Table 9**). These proteins were associated with, among others, such biological processes as *movement in host environment* (11 DRPs; GO:0052126), *interaction with host* (12 DRPs; GO:0051701), *entry into host* (10 DRPs; GO:0044409) or *adhesion of symbiont to host* (2 DRPs; GO:0044406). Pathways enrichment analysis revealed 26 metabolic pathways involving 104 of the 161 proteins analyzed (**Figure 2H**, **Supplementary Table 10**). These included *biosynthesis of amino acids* (8 DRPs; hsa01230), *carbon metabolism* (7 DRPs; hsa01200), *oxidative phosphorylation* (9 DRPs; hsa00190), or *glycolysis/gluconeogenesis* (5 DRPs; hsa00010).

The GO analysis of DRPs identified in Caco-2 after treatment with high concentration of extracellular vesicles (Anis-EVs High) showed 484 BPs, 108 CCs, and 106 MFs (**Figure 2I**, **Supplementary Table 11**). Processes such as *viral transcription* (16 DRPs; GO:0019083), *viral gene expression* (16 DRPs; GO:0019080), *interaction with host* (12 DRPs; GO:0051701), *interaction with symbiont* (5 DRPs; GO: 0051702) or *modulation by host of symbiont process* (4 DRPs; GO:0051851) were noted. Pathway enrichment analysis revealed 35 metabolic pathways, in which, in addition to those noted after culturing Caco-2 cells with *Anisakis* larvae or after treating cells with Anis-EV Low, the following were also observed: *cholesterol metabolism* (7 DRPs; hsa04979), *necroptosis* (10 DRPs; hsa04217), *thermogenesis* (10 DRPs; hsa04714), or *chemical carcinogenesis - reactive oxygen species* (14 DRPs; hsa05208) (**Figures 2H**, **Supplementary Table 12**).

### 3.4 DRPs establish a complex network of interactions in host cells

**Figure 3:**
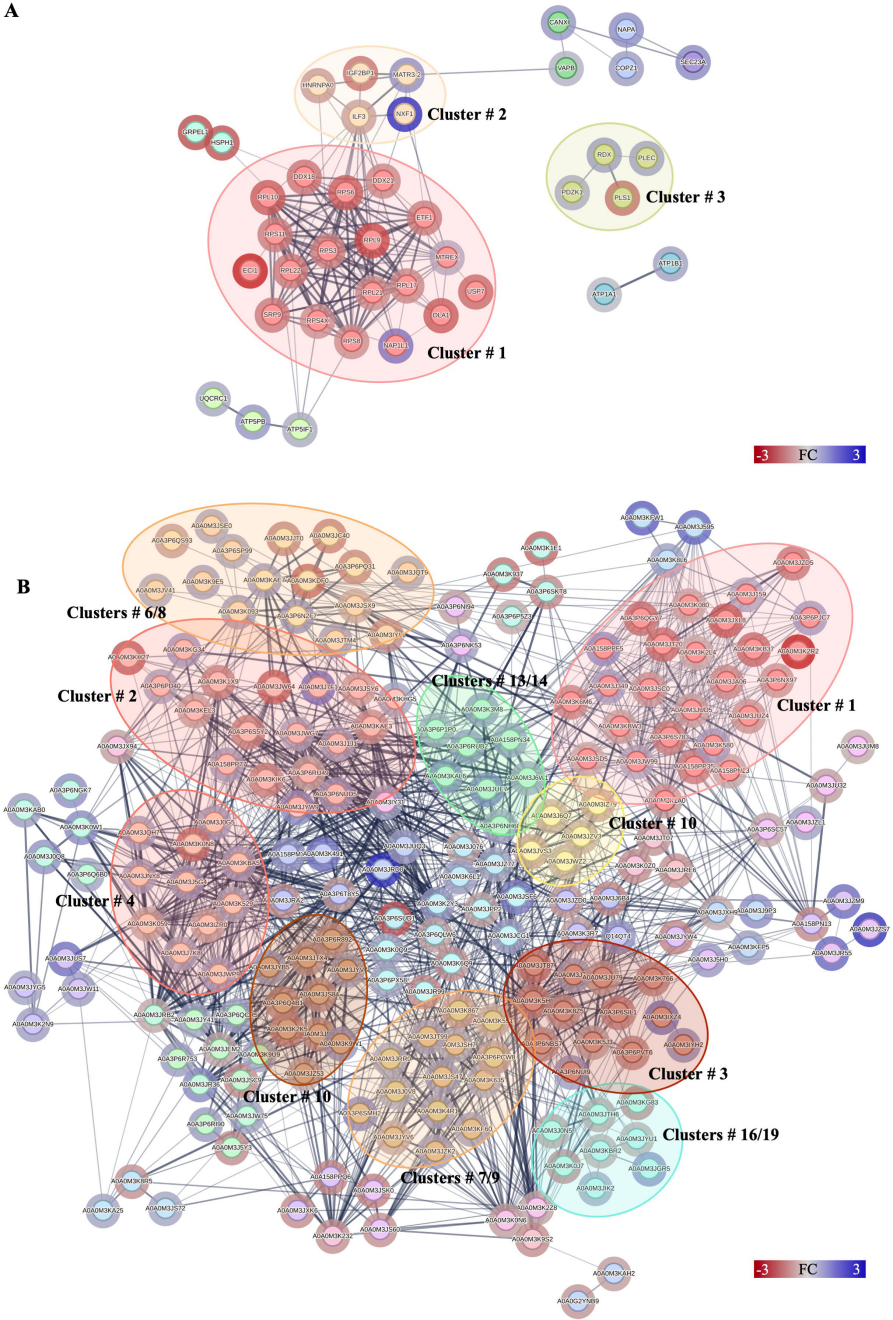
Protein–protein interactions network analysis of DRPs identified in this study. Interactions of DRPs identified in Caco-2 cells after **(A)** co-culture with *A*. *simplex* (s. s.) larvae, and **(B)** interactions between DRPs identified in *A*. *simplex* (s. s.) larvae after co-culture with Caco-2 cells. Red circle borders – up-, green circle borders – downregulated proteins. The Markov Cluster Algorithm (MCL) was used for the clustering the network (inflation parameter = 3). Different colors inside the circles are different clusters. Selected clusters are described by numbers in the figure. The results of statistical analysis are present in **Supplementary Table 13**. Detailed information about the number of interactions and clusters’ descriptions can be found in the **Supplementary Tables 14, 15, 23** and **24**.

**Figure 4:**
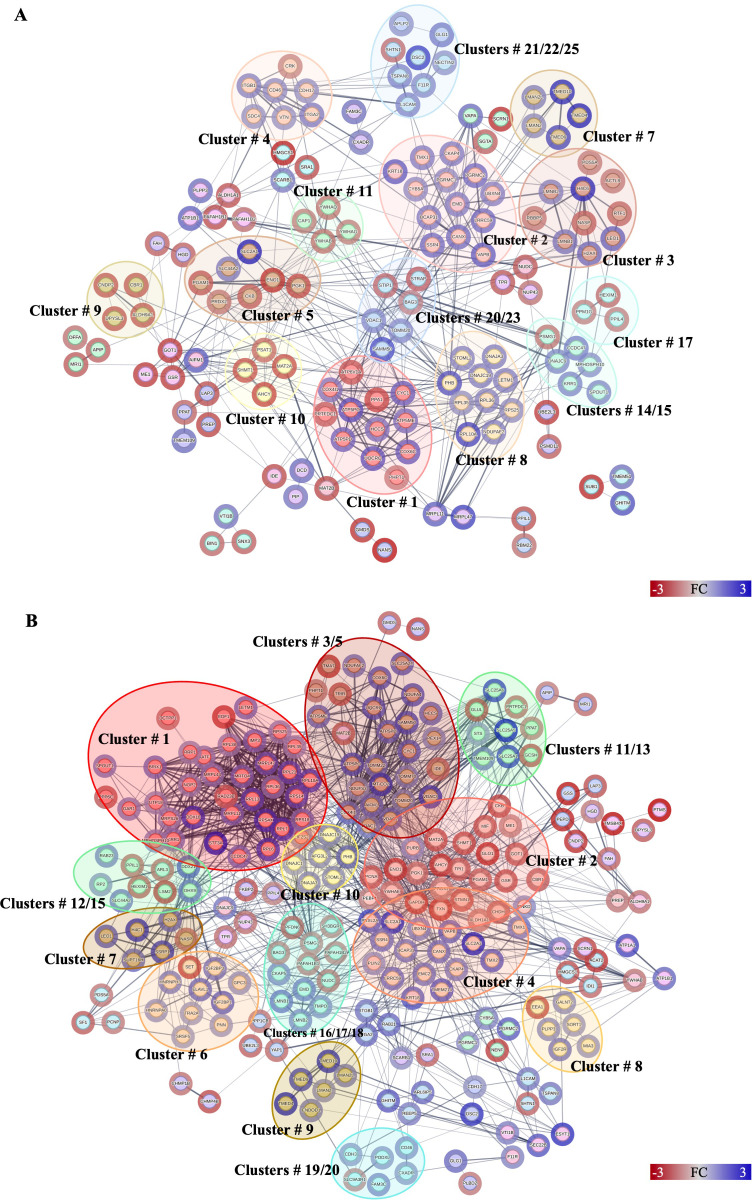
Protein–protein interactions network analysis of DRPs identified in this study. Interactions of DRPs identified in Caco-2 cells after treatment with **(A)** low, and **(B)** high concentration of Anis-EVs. Red circle borders – up-, green circle borders – downregulated proteins. The Markov Cluster Algorithm (MCL) was used for the clustering the network (inflation parameter = 3). Different colors inside the circles are different clusters. Selected clusters are described by numbers in the figure. The results of statistical analysis are present in **Supplementary Table 13**. Detailed information about the number of interactions and clusters’ descriptions can be found in the **Supplementary Tables 16-19**.

The network of protein interactions was performed by submitting only DRPs to Cytoscape (v. 3.8.0.; NIGMS, Bethesda, MD, USA), and analyzed by stringApp (v. 1.5.1.) (47). The visualizations demonstrated strong interactions networks (**Figures 3**, **4**).

Forty proteins, out of 49 DRPs in Caco-2 cells co-cultured with *A. simplex* (s. s.) larvae, constituted complex network with 146 interactions (**Figure 3A**). The *small ribosomal subunit protein uS3* (RPS3) had the highest number of interactions with other proteins (18 interactions) (**Supplementary Table 14**). MCL clustering revealed 9 groups of proteins, one of which contained as many as 19 interacting proteins (**Figure 3A**, cluster no. 1). This cluster grouped proteins involved in the process of protein translation (**Supplementary Table 15**).

A total of 146 DRPs, out of 165 DRPs identified in Caco-2 cells treated with Anis-EVs Low, constituted a very complex and strongly interactive network (456 interactions) (**Figure 4A**, **Supplementary Table 13**). The proteins with the highest number of interactions with other proteins were *calnexin* (CANX; 28 interactions), *voltage-dependent anion-selective channel protein* (VDAC1; 28 interactions), *prohibitin* (PHB; 26 interactions), and *alpha-enolase* (ENO1; 25 interactions) (**Supplementary Table 16**). MCL clustering revealed 45 clusters (**Supplementary Table 17**). These clusters included proteins involved in processes such as oxidative phosphorylation (cluster no. 1), beta-catenin formation (cluster no. 3), glycolysis/gluconeogenesis (cluster no. 7) or cysteine and methionine metabolism (cluster no. 10) (**Figure 4A**, **Supplementary Table 17**).

Among the DRPs identified in Caco-2 cells after treatment with Anis-EVs High (233 out of a total of 256 DRPs) (**Figure 4B**, **Supplementary Table 13**), the proteins that had the highest number of interactions were *glyceraldehyde-3-phosphate dehydrogenase* (GAPDH; 72 interactions), *prohibitin* (PHB; 46 interactions), *40S ribosomal protein S18* (RPS18; 43 interactions), *calnexin* (CANX; 41 interactions), and *triosephosphate isomerase* (TPI1; 40 interactions) (**Figure 4B**, **Supplementary Table 18**). In this group, the MCL algorithm showed 49 clusters. The largest of these grouped proteins involved in processes such as nuclear and cytosolic rRNA processing and translation (cluster no. 1), amino acid biosynthesis (cluster no. 2) and mitochondria-associated membrane processes of the endoplasmic reticulum (cluster no. 4). Clusters 3 and 5 grouped proteins responsible for oxidative phosphorylation and protein incorporation into the outer mitochondrial membrane, respectively (**Figure 4B**, **Supplementary Table 19**).

The statistical results from STRING analysis, the node degrees (number of interactions), and the results of MCL clustering of DRPs identified in Caco-2 after co-culture with *A. simplex* (s. s.) larvae and treatment with Anis-EVs can be found in **Supplementary Tables 13, 16**–**19**.

### 3.5 Anisakis simplex larvae reacted to co-culture with host cells with changes in their proteome

**Figure 5:**
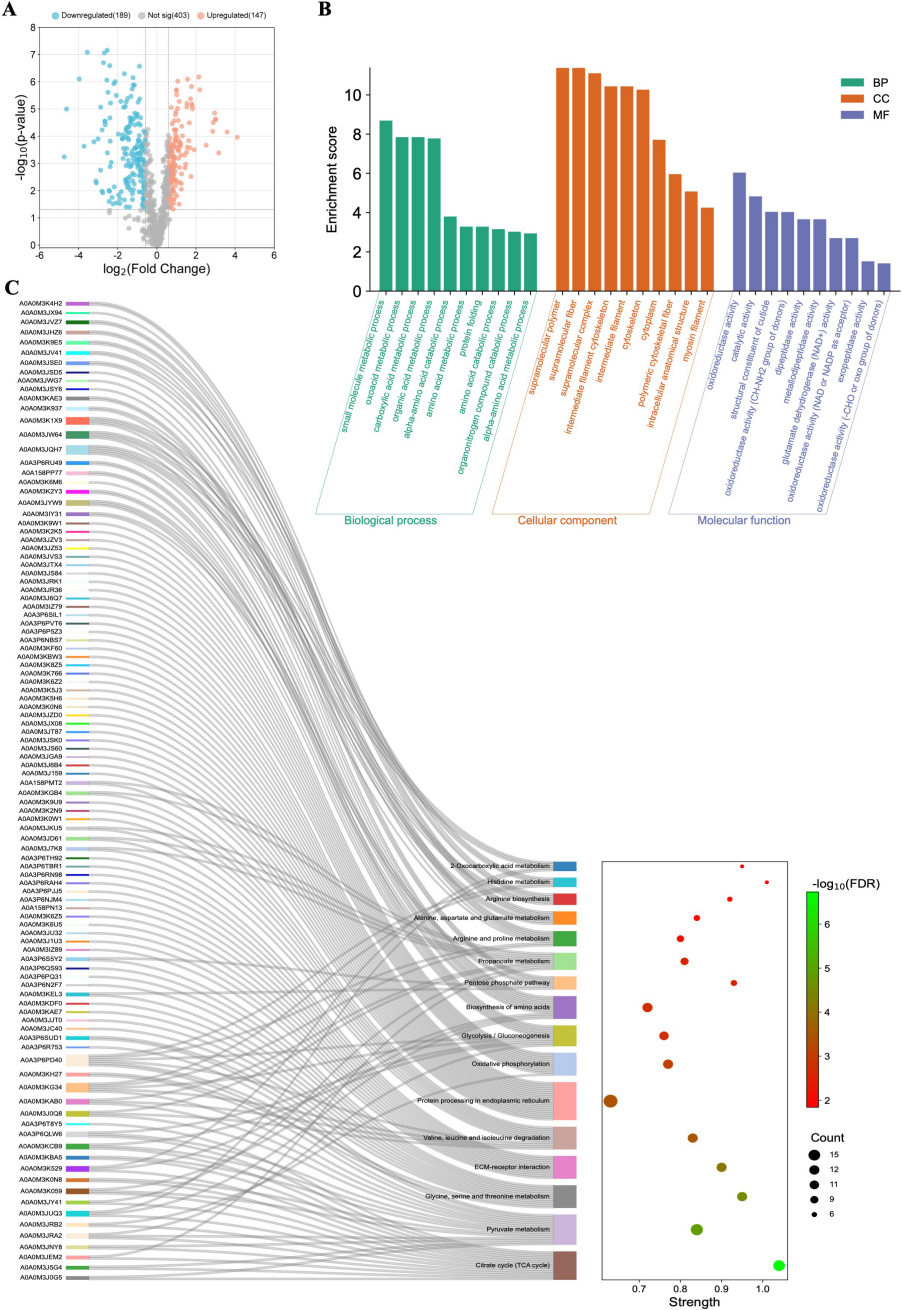
Functional characterization of the differentially regulated proteins (DRPs) identified in *Anisakis simplex* (s. s.) larvae after co-culture with Caco-2 cells. **(A)** Volcano-plot representation of identified DRPs. FC = 1.5; *p*-value ≤ 0.05. **(B)** The visualization of GO analysis results and **(C)** pathways enrichment analysis results. Detailed results can be found in the **Supplementary Tables 21** and **22**.

In addition, we also examined the proteomic response of *A. simplex* (s. s.) larvae when they were co-cultured with Caco-2 cells. We found that a total of 336 proteins were differently regulated in the larvae co-cultured with Caco-2 compared to the control group. Out of these, 147 proteins were upregulated, and 189 proteins were downregulated (**Figure 5A**). The specific proteins identified in this comparison can be found in **Supplementary Table 20**.

DRPs identified in *A. simplex* (s. s.) larvae after co-culture with Caco-2 cells were also subjected to GO analysis. The co-culture with cells influenced the differential regulation of 336 proteins, which resulted in 27 BPs, 23 CCs, and 12 MFs (**Supplementary Table 21**). The top ten significant GO terms are shown in **Figure 5B**. Proteins classified as DRPs were also subjected to pathway enrichment analysis, which indicated their involvement in 19 metabolic pathways, including *citrate cycle (TCA cycle)* (15 DRPs; map00020), *pyruvate metabolism* (16 DRPs; map00620), *ECM-receptor interaction* (12 DRPs; map04512), *glyoxylate and dicarboxylate metabolism* (21 DRPs; map00630), or *oxidative phosphorylation* (12 DRPS; map00190) (**Figure 5C**, **Supplementary Table 22**).

The network formed by 212 DRPs, out of a total of 336 DRPs identified in *Anisakis* larvae after co-culture with Caco-2 cells, showed 1,403 interactions (**Figure 3B**). The highest number of interactions was observed by *glyceraldehyde-3-phosphate dehydrogenase* (A0A0M3K2Y3; 57 interactions), *malate* dehydrogenase (A0A0M3KBA5; 56 interactions), *PPIase cyclophilin-type domain-containing protein* (A0A3P6RU49; 51 interactions), *aconitate hydratase, mitochondrial* (A0A0M3JUQ3; 49 interactions), *fatty acid-binding protein homolog 9* (A0A0M3J6W1; 45 interactions) or *succinate dehydrogenase [ubiquinone] iron-sulfur subunit*, mitochondrial (A0A0M3JRB2; 40 interactions) (**Figure 3B**, **Supplementary Table 23**). The analysis of protein-protein interactions with MCL clustering revealed 51 clusters. The largest of these are shown in **Figure 3B**. All proteins contained in the individual clusters can be found in **Supplementary Table 24**.

### 3.6 The cytokine profile of Caco-2 cells under the influence of Anis-EVs is anti-inflammatory

**Figure 6:**
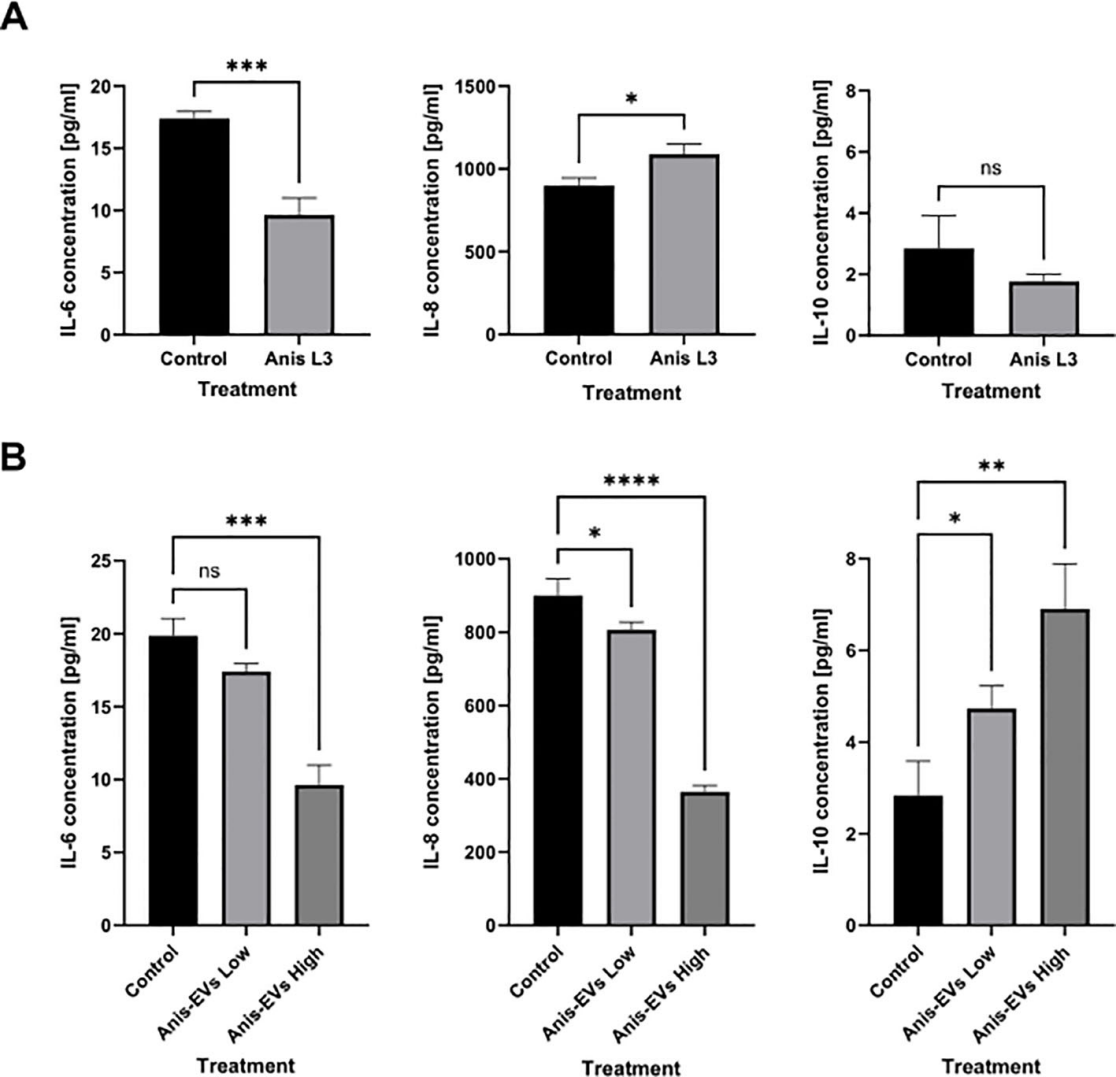
Cytokines secretion by Caco-2 cells. The secretion of IL-6, IL-8, and IL-10 into the culture medium by Caco-2 cells under the influence of **(A)** co-culture with *A*. *simplex* (s. s.) larvae and **(B)** after treatment with different concentrations of Anis-EVs (Anis-EVs Low – ~500 particles per cell and Anis-EVs High – ~5,000 particles per cell). Ordinary one-way ANOVA analysis was performed and the differences between means were assessed by Dunnett’s multiple comparisons test. Values were considered statistically significant, where 0.0332 (∗), 0.0021 (∗∗), 0.0002 (∗∗∗), and <0.0001 (∗∗∗∗). ns, non-significant results.

The secretion of cytokines by Caco-2 cells into the culture medium was investigated to determine the pro-inflammatory or anti-inflammatory effect of *A. simplex* (s. s.) L3 larvae or Anis-EVs (**Figure 6**).

Treatment with the *A. simplex* (s. s.) L3 larvae resulted in a decrease in IL-6 concentration in the culture medium (*p* = 0.0008) compared to the non-treated cells (**Figure 6A**). The concentration of IL-8 in the culture medium was increased after treatment with the live L3 larvae (*p* = 0.0127). In contrast, the concentration of the anti-inflammatory cytokine IL-10 showed no significant changes (**Figure 6A**).

Culture with Anis-EVs led to a progressive decrease in pro-inflammatory IL-6 and IL-8 concentrations in the medium collected after Caco-2 culture (*p* < 0.05) (**Figure 6B**). In addition, the presence of Anis-EVs increased the concentration of the potent anti-inflammatory IL-10 (p < 0.05) (**Figure 6B**). In all cases, the more pronounced changes were caused by a higher concentration of extracellular vesicles (Anis-EVs High; ~5,000 particles per cell).

## 4 Discussion

Parasitic nematodes have long been recognized as formidable enemies of human and animal health, causing a variety of diseases worldwide. A fascinating aspect of their parasitic lifestyle is their ability to manipulate host physiology through intricate molecular mechanisms. Recent research has shed light on the role of extracellular vesicles (EVs) in mediating host-parasite interactions (18). These nano-sized membrane-bound vesicles, secreted by a variety of parasitic nematodes, serve as effective vehicles for intercellular communication. Understanding the composition and function of nematode-derived EVs opens new avenues for exploring the complex interplay between parasites and their hosts, which has profound implications for disease pathogenesis and therapeutic interventions (24, 48).

In the present study, *A. simplex* (s. s.) infection of the human host was simulated *in vitro* using human Caco-2 cells and whole *A. simplex* (s. s.) larvae or Anis-EVs. The study is the first attempt to use a multiproteomic approach to investigate the molecular mechanism of anisakiasis, i.e. the active invasion of the host intestinal tissue by parasitic larvae and the direct influence of EVs as recently recognized messengers of pathogenicity.

So far, the proteomic profiling of secretome of *A. simplex* has been described by Kochanowski et al. (49). The aim of study was to identify and characterize the excretory-secretory (ES) proteins of *A. simplex* L3 larvae. However, these were not proteins directly derived from EVs, but the general ES proteome. Recently, Boysen et al. (50) performed proteomic analysis of *Anisakis* spp.-derived EVs and showed the presence of 15 different proteins in isolated vesicles. In this study, we detected the presence of 23 different annotated proteins in Anis-EVs (e.g., Argonaute-1, RNA-dependent RNA polymerase, nematode eukaryotic translation initiation factor 3, or transposase). The sets of proteins identified by Boysen et al. (50) and in this study do not overlap, which may be due to the different *in vitro* conditions, which could have resulted in differences in the cargo of vesicles from the same species. Moreover, in both cases the larvae were from different populations of nematodes (different geographical regions). Vesicle contents have also been found to differ depending on gender of the helminth, what was not checked in both studies (51, 52). Furthermore, the methods used to isolate the EVs may have influenced this. However, the most common technique for isolating and purifying EVs from parasitic helminths is ultracentrifugation at 100,000 x g or more for at least (32, 53).

The few proteins identified in Anis-EVs play a central role in RNA silencing processes, justifying their impact on host cells compared to whole larvae (54, 55) (**Figures 2A–C**). Two proteins attract particular attention: Argonaute-1 (A0A0M3IZ63) and RNA-dependent RNA polymerase (A0A0M3KE70). Small RNAs are bound by Argonaute proteins which take role in RNA-loading into EVs (56). Gastrointestinal parasites such as *Heligmosomoides bakeri* require a mechanism to transport specific small RNAs into host cells (mammalian cells) (57). Packaging of secondary small interfering RNAs (siRNAs) into EVs by extracellular worm Argonautes (WAGOs or ‘WA-GOs’) provides a key mechanism for export selectivity. It is not yet known whether extracellular WAGOs are only involved in export or also in mediating functional effects within the recipient cells. Several studies suggest that mammalian Argonautes can be very stable in extracellular environments outside EVs, but whether and how they are internalized by cells is not known (55). Furthermore, Chow et al. (55) recently stated that the most abundant small RNAs released from the parasitic nematode are not microRNAs as previously thought, but siRNAs that are produced by RNA-dependent RNA Polymerases, and such polymerase has been detected in Anis-EVs in this study. In addition, eukaryotic translation initiation factor 3 (eIF3, subunit L; A0A158PN31) and mRNA (guanine-N (7)) methyltransferase (RNMT; A0A3P6PCH2), which are involved in the maturation and translation of mRNA in eukaryotes (58, 59), as well as transposase (A0A0M3JEA3), which is capable of transposon movement to another part of genome (60), have been identified in Anis-EVs.

Recently, the protein repertoire associated with EVs in *Anisakis pegreffii*, a sibling species of *A. simplex* (s. s.), was characterized using a *de novo* transcriptome assembly (61). The authors of this study identified 153 proteins. Such a different number of identified proteins could also be due to the fact that the EVs isolated by Palomba et al. (61) were less homogeneous than the Anis-EVs considering the results of the NTA and TEM analyses, which could be reflected in a higher number of packaged proteins. The characterization of the proteins in the EVs enriched with *A. pegreffii* showed that they may be involved in the survival and adaptation of the parasite as well as in pathogenic processes (61). The protein repertoire associated with *A. pegreffii* did not contain identical proteins compared to Anis-EVs, but similar proteins whose functions also consisted in the regulation of transcription and gene expression, e.g., elongation factor 1-alpha/gamma (61).

The proteins of Anis-EVs do not show much similarity compared to other ascaridoid nematodes, i.e. *Ascaris suum* (62), *Brugia malayi* (52), *Nippostrongylus brasiliensis* (63) and *Trichuris muris* (64). However, proteins such as eukaryotic initiation factors (eIFs), which are found in the vesicles of *A. suum* and *T. muris*, were also found in Anis-EVs. Nevertheless, further work is needed to understand the individual and collective contributions of all EVs cargos to the immunomodulation in the host recipient cells.

The aim of this study was also to analyze the effects of Anis- EVs compared to whole L3 larvae of *A. simplex* (s. s.) on the proteome of the human intestinal model, Caco-2 cells. This cell line has already been successfully used to study the interactions between different pathogens and the human host and is considered the gold standard for this type of *in vitro* studies (65–70).

The results of the research conducted clearly indicate that *A. simplex* (s. s.) can induce a stronger proteomic response in the host organism by EVs alone than by whole larvae. In all cases examined, the proteome of Caco-2 cells was altered by the interaction with Anis-EVs or L3 larvae (**Figure 2**). However, the greatest differences compared to the control were observed when Caco-2 was treated with a high concentration of Anis-EVs. Similarly, studies by Bellini et al. (66) showed that Caco-2 cells were not significantly triggered by the presence of live L3 larvae of *Anisakis* spp. In addition, our results of pro-inflammatory cytokine assays showed a decrease in IL-6 levels and an increase in IL-8 levels when Caco-2 was co-cultured with L3 larvae. It is hypothesized that these specific cytokine levels decrease due to the parasite’s strategy to modulate the epithelial barrier response triggered by initial contact with the host to rapidly return the system to hemostasis and keep the host healthy for successful long-term invasion (71). As there were no statistical differences in the results on IL-10 levels, it is not possible to clearly determine the nature (pro- or anti-inflammatory) of the direct interaction of Caco-2 cells with *A. simplex* (s. s.) L3 larvae.

The available *in vitro* studies describe a pro-inflammatory effect of *Anisakis* crude extract, which is a mixture of endotoxins, proteases, and somatic proteins, including upregulation of oxidative stress, alteration of barrier integrity, inhibition of apoptosis-related biomarkers and induction of inflammation in host (13, 72–74). The results obtained here indicate the potential anti-inflammatory effect of Anis-EVs on Caco-2 recipient cells. In the study, two concentrations of Anis-EVs were used to simulate moderate (Anis-EVs Low) and intensive (Anis-EVs High) invasion. Tests on the levels of pro- and anti-inflammatory cytokines secreted by Caco-2 under the influence of Anis-EVs showed in all cases the more pronounced changes caused by a higher concentration of EVs (Anis-EVs High). In the present study, a consistent result was obtained on the suppressive effect of Anis- EVs, showing the decrease in the level of the pro-inflammatory cytokines IL-6 and IL-8 and the increase in the level of the anti-inflammatory cytokine IL-10. Similar conclusions based on IL-6 and IL-8 levels were reached by Bellini et al. (66). Moreover, authors of the study suggested that the decrease in these pro-inflammatory cytokines could be via potential regulatory factors such as miRNAs, which are also transported in EVs. Eichenberger et al. showed that EVs from the *N. brasiliensis* protected against intestinal inflammation in mice, resulting in suppression of pro-inflammatory cytokines IFN-γ, IL-6, IL-17a, and IL-1β. Moreover, *N. brasiliensis* EVs promoted the production of IL-10, suggesting a potential mechanism of systemic regulation of inflammation in a mouse model of inducible chemical colitis (63). Accordingly, infective stages (L3) of *B. malayi*, a filarial parasite responsible for human lymphatic filariasis, release EVs that interact *in vitro* with a murine macrophage cell line and stimulate classical (pro-inflammatory) activation of macrophages (75). However, the immunomodulatory capacity of EVs from a parasitic nematode of the murine gastrointestinal tract, *H. polygyrus*, has been demonstrated by suppressing innate type 2 immune responses (76).

It has been suggested that EVs play a role in the host-parasite communication and, subsequently, in the development of the infection and in the evasion of the host immunological response (22–25). However, based on the results of these and other studies, the molecular basis of the anti-inflammatory or pro-inflammatory nature of EVs on the host organism cannot be clearly determined. Nematodes therefore most likely use a precisely balanced mixture of soluble and vesicular molecules to control the host’s immunity and thus evade the host’s immune system.

The proteomic analysis of Caco-2 cells cultured with a low dose of Anis-EVs showed that 165 proteins were differentially regulated compared to the control. On the other hand, analysis of Caco-2 cells cultured with a high dose of EVs showed 256 DRPs. These proteins in both cases were associated with, among others, such biological processes as *movement in host environment*, *interaction with host*, *interaction with symbiont*, *entry into host* or *adhesion of symbiont to host*.

The proteins involved in the biological processes mentioned, including insulin-degrading enzyme (IDE), sorting nexin-3 (SNX3), prohibitin (PHB), integrins alpha-2 and beta-1 (ITGA2, ITGB1), vesicle-associated membrane protein-associated proteins (VAPB/C and VAPA), junctional adhesion molecule A (JAM-A), membrane cofactor protein (MCP/CD46), SCARB1 protein, nectin-2, charged multivesicular body protein 4b (CHMP4B) and glyceraldehyde-3-phosphate dehydrogenase (GAPDH) suggest several mechanisms by which nematode EVs may influence host cellular functions and contribute to infection and pathogenesis.

Modulation of IDE can disrupt insulin metabolism, leading to altered glucose homeostasis, which may create a favorable environment for parasite survival by impairing cellular energy balance (77). Alterations in SNX, CHMP4B and VAPs indicate possible disruption of endosomal sorting and vesicle transport, which are critical for maintaining cellular signaling, protein recycling and immune response, thereby aiding in immune evasion and creating a more favorable environment for the parasite. In addition, recent studies on one of the SNX confirmed its involvement as a regulator of anti-inflammatory response in experimental mouse colitis (78, 79). Integrin alpha-2 and beta-1, as well as adhesion molecules such as JAM-A and nectin-2, are critical for maintaining the epithelial barrier and facilitating cell-cell interactions. Alterations in these proteins can weaken cellular junctions, which increases tissue permeability and promotes nematode migration (80–83). In addition, the alteration of MCP/CD46, a regulator of the complement system, suggests a mechanism to evade the immune system by some strategy to overcome CD46-host mediated cytoprotection (84). PHB play essential role in mitosis and cell proliferation and its modulation has been described in human foreskin fibroblasts during *Toxoplasma gondii* infection (85). GAPDH is used to break down glucose to produce energy and carbon molecules during the sixth step of glycolysis. It is also known for its functions including the cytoskeletal organization, DNA repair mechanisms, apoptosis and ROS-mediated oxidative scavenging (86). Its modulation could alter glucose homeostasis to provide a favorable environment for the parasite and promote its persistence. Recently, GAPDH has also been proposed as a suitable vaccine candidate for protection against parasitic diseases (87). However, the homology between the host and pathogen GAPDH proteins is currently an insurmountable obstacle.

Metabolic pathways identified in the analysis of the KEGG database as enriched in Caco-2 cells after treatment with Anis-EVs, such as oxidative phosphorylation, glycolysis/gluconeogenesis, biosynthesis of amino acids, protein processing in the endoplasmic reticulum, ECM receptor interaction, phagosome or pathways related to cell adhesion molecules, also clearly confirm the involvement of the above-mentioned proteins and indicates that EVs play an important role in host-parasite cross-talk in *A. simplex* (s. s.) infection.

We were also able to demonstrate the proteomic response of *A. simplex* (s. s.) larvae to host cells after *in vitro* co-culture. To our knowledge, such an experiment has never been performed before. The results of the proteome analysis of *A. simplex* (s. s.) larvae indicate changes in the activity of metabolic pathways such as the citrate cycle (TCA cycle), pyruvate metabolism, oxidative phosphorylation or glycolysis/gluconeogenesis. Particularly noteworthy are metabolic pathways such as ECM-receptor interaction, protein processing in the endoplasmic reticulum or biosynthesis of amino acids, which indicate enriched protein metabolic processes. GO analysis of biological processes also indicates changes in protein metabolism (amino acid metabolic processes and protein folding). In addition, GO analysis showed that the DRPs identified in *A. simplex* (s. s.) larvae are characterized by oxidoreductase activity, catalytic activity and peptidase activity, i.e. proteins responsible for invasion, including facilitating penetration of host tissues or protecting the parasite from host immune system attack, as they are able to degrade immunoglobulins (88–90) (**Supplementary Table 21**).

Furthermore, such activity in protein metabolism could also be related to the biogenesis of EVs, but we do not yet know a direct answer to the question of the source and means of biosynthesis of helminth EVs, as well as to the broader question of what their precise physiological function might be (51).

## 5 Conclusions

The results of this study indicate the potential anti-inflammatory character of Anis-EVs in relation to Caco-2 cells. At the same time, direct treatment with Anis-EVs resulted in more significant changes in the Caco-2 proteome than co-culture with *A. simplex* (s. s.) L3 larvae. The proteomic profile of *A. simplex* (s. s.) L3 larvae also changed after co-culture with Caco-2 cells.

Our understanding of the mechanisms of EVs biogenesis pathways in parasites, particularly in parasitic nematodes, is still at an early stage. Nevertheless, continued growth in this area will enable a better understanding of infection mechanisms and immune responses, as well as the development of new diagnostic tools and vaccines. However, all efforts should now focus on defining the composition of EVs, the exact cells that produce them and the metabolic pathways that are affected by them in the host organism.

The results obtained in *A. simplex* (s. s.) should lead to a better understanding of the molecular processes underlying the development of this parasite infection in humans and will add to the existing knowledge of the role of EVs in host-parasite communication.

The future integration of proteomic data with other omics datasets, such as transcriptomics and metabolomics, will improve our understanding of the complex networks that govern host-parasite interactions and pave the way for the development of innovative strategies to combat parasitic diseases. The better description of EVs functions in nematodes have the potential both to transform our understanding of parasite adaptation to the host and to develop possible therapies for immune-mediated diseases.

We should be aware that there is a great heterogeneity of EVs, which may depend on many factors, such as the developmental stage of the nematodes, their sex or the time after host invasion. Characterizing the cell and tissue status of the host during the invasion of these EVs may lead to a better understanding of EV functions in host-parasite communication. Therefore, the future direction in the studies on *A. simplex* (s. s.) will be to characterize the transcriptomic and metabolomic cargo of EVs produced by this parasitic nematode as well as to describe the host omic response at the tissue levels to determine host-parasite interactions more precisely, to which the recently popular host-derived organoids may contribute significantly.

## Data Availability

The datasets presented in this study can be found in online repositories. The names of the repository/repositories and accession number(s) can be found below: MSV000092443 (MassIVE repository; www.massive.ucsd.edu; https://massive.ucsd.edu/ProteoSAFe/dataset.jsp?task=264259724f8440fcbc35cf624d914c33).

## References

[1] Mattiucci S Cipriani P Levsen A Paoletti M Nascetti G. Molecular epidemiology of anisakis and anisakiasis: an ecological and evolutionary road map. Adv Parasitol. (2018) 99:93–263. doi: 10.1016/bs.apar.2017.12.001 29530312

[2] Ivanović J Baltić MŽ Bošković M Kilibarda N Dokmanović M Marković R. Anisakis allergy in human. Trends Food Sci Technol. (2017) 59:25–9. doi: 10.1016/j.tifs.2016.11.006

[3] Shimamura Y Muwanwella N Chandran S Kandel G Marcon N. Common symptoms from an uncommon infection: gastrointestinal anisakiasis. Can J Gastroenterol Hepatol. (2016) 2016:1–7. doi: 10.1155/2016/5176502 PMC5075291 27800471

[4] Audicana MT Ansotegui IJ de Corres LF Kennedy MW. Anisakis simplex: dangerous — dead and alive? Trends Parasitol. (2002) 18:20–5. doi: 10.1016/S1471-4922(01)02152-3 11850010

[5] Nieuwenhuizen NE. Anisakis - immunology of a foodborne parasitosis. Parasite Immunol. (2016) 38:548–57. doi: 10.1111/pim.12349 27428817

[6] Nieuwenhuizen NE Lopata AL. Allergic reactions to anisakis found in fish. Curr Allergy Asthma Rep. (2014) 14:455. doi: 10.1007/s11882-014-0455-3 25039016

[7] EFSA. Scientific Opinion on risk assessment of parasites in fishery products. EFSA J. (2010) 8:1543. doi: 10.2903/j.efsa.2010.1543

[8] Klimpel S Palm HW. Anisakid Nematode (Ascaridoidea) Life Cycles and Distribution: Increasing Zoonotic Potential in the Time of Climate Change? In: Mehlhorn H, editor. Progress in Parasitology. Parasitology Research Monographs. Springer Berlin Heidelberg, Berlin, Heidelberg (2011). p. 201–22. doi: 10.1007/978-3-642-21396-0_11

[9] Schopf LR Hoffmann KF Cheever AW Urban JF Wynn TA. IL-10 Is critical for host resistance and survival during gastrointestinal helminth infection. J Immunol. (2002) 168:2383–92. doi: 10.4049/jimmunol.168.5.2383 11859129

[10] Audicana MT Kennedy MW. Anisakis simplex: from obscure infectious worm to inducer of immune hypersensitivity. Clin Microbiol Rev. (2008) 21:360–79. doi: 10.1128/CMR.00012-07 PMC2292572 18400801

[11] Hepworth MR Grencis RK Artis D. Regulation of immunity and inflammation following intestinal helminth infection. In: Parasitic nematodes: molecular biology, biochemistry and immunology. Wallingford: CABI, Wallingford, UK (2013). p. 106–29. doi: 10.1079/9781845937591.0106

[12] Garcia-Perez JC Rodríguez-Perez R Ballestero A Zuloaga J Fernandez-Puntero B Arias-Díaz J. Previous exposure to the fish parasite anisakis as a potential risk factor for gastric or colon adenocarcinoma. Medicine. (2015) 94:e1699. doi: 10.1097/MD.0000000000001699 26448021 PMC4616760

[13] Speciale A Trombetta D Saija A Panebianco A Giarratana F Ziino G. Exposure to Anisakis extracts can induce inflammation on *in vitro* cultured human colonic cells. Parasitol Res. (2017) 116:2471–7. doi: 10.1007/s00436-017-5551-6 28702801

[14] Raybourne R Deardorff TL Bier JW. Anisakis simplex: Larval excretory secretory protein production and cytostatic action in mammalian cell cultures. Exp Parasitol. (1986) 62:92–7. doi: 10.1016/0014-4894(86)90012-3 3720903

[15] Baird FJ Gasser RB Jabbar A Lopata AL. Foodborne anisakiasis and allergy. Mol Cell Probes. (2014) 28:167–74. doi: 10.1016/j.mcp.2014.02.003 24583228

[16] Koutsoumanis K Allende A Alvarez-Ordóñez A Bover-Cid S Chemaly M De Cesare A. Re-evaluation of certain aspects of the EFSA Scientific Opinion of April 2010 on risk assessment of parasites in fishery products, based on new scientific data. Part 1: ToRs1–3. EFSA J. (2024) 22(4):e8719. doi: 10.2903/j.efsa.2024.8719 38650612 PMC11033839

[17] Estevez AM Kempf T Clayton C. The exosome of Trypanosoma brucei. EMBO J. (2001) 20:3831–9. doi: 10.1093/emboj/20.14.3831 PMC125547 11447124

[18] Marcilla A Martin-Jaular L Trelis M de Menezes-Neto A Osuna A Bernal D. Extracellular vesicles in parasitic diseases. J Extracell Vesicles. (2014) 3:25040. doi: 10.3402/jev.v3.25040 25536932 PMC4275648

[19] Doyle L Wang M. Overview of extracellular vesicles, their origin, composition, purpose, and methods for exosome isolation and analysis. Cells. (2019) 8:727. doi: 10.3390/cells8070727 31311206 PMC6678302

[20] Bobrie A Colombo M Raposo G Théry C. Exosome secretion: molecular mechanisms and roles in immune responses. Traffic. (2011) 12:1659–68. doi: 10.1111/j.1600-0854.2011.01225.x 21645191

[21] Maas SLN Breakefield XO Weaver AM. Extracellular vesicles: unique intercellular delivery vehicles. Trends Cell Biol. (2017) 27:172–88. doi: 10.1016/j.tcb.2016.11.003 PMC5318253 27979573

[22] Marcilla A Trelis M Cortés A Sotillo J Cantalapiedra F Minguez MT. Extracellular vesicles from parasitic helminths contain specific excretory/secretory proteins and are internalized in intestinal host cells. PloS One. (2012) 7(9):e45974. doi: 10.1371/journal.pone.0045974 23029346 PMC3454434

[23] Buck AH Coakley G Simbari F McSorley HJ Quintana JF Le Bihan T. Exosomes secreted by nematode parasites transfer small RNAs to mammalian cells and modulate innate immunity. Nat Commun. (2014) 5:1–11. doi: 10.1038/ncomms6488 PMC4263141 25421927

[24] Coakley G Maizels RM Buck AH. Exosomes and other extracellular vesicles: the new communicators in parasite infections. Trends Parasitol. (2015) 31:477–89. doi: 10.1016/j.pt.2015.06.009 PMC4685040 26433251

[25] Riaz F Cheng G. Exosome-like vesicles of helminths: Implication of pathogenesis and vaccine development. Ann Transl Med. (2017) 5:10–2. doi: 10.21037/atm.2017.03.45 PMC5401686 28480211

[26] Hotez PJ Brindley PJ Bethony JM King CH Pearce EJ Jacobson J. Helminth infections: the great neglected tropical diseases. J Clin Invest. (2008) 118:1311–21. doi: 10.1172/JCI34261 PMC2276811 18382743

[27] Toledo R Bernal MD Marcilla A. Proteomics of foodborne trematodes. J Proteomics. (2011) 74:1485–503. doi: 10.1016/j.jprot.2011.03.029 21459168

[28] Niciura SCM Cardoso TF Ibelli AMG Okino CH Andrade BG Benavides MV. Multi-omics data elucidate parasite-host-microbiota interactions and resistance to Haemonchus contortus in sheep. Parasit Vectors. (2024) 17:102. doi: 10.1186/s13071-024-06205-9 38429820 PMC10908167

[29] Tort JF Mitreva M Brehm KR Rinaldi G. Editorial: novel frontiers in helminth genomics. Front Genet. (2020) 11:791. doi: 10.3389/fgene.2020.00791 32922432 PMC7456986

[30] Chu X Zhang B Koeken VACM Gupta MK Li Y. Multi-omics approaches in immunological research. Front Immunol. (2021) 12:668045. doi: 10.3389/fimmu.2021.668045 34177908 PMC8226116

[31] Petriz BA Franco OL. Metaproteomics as a complementary approach to gut microbiota in health and disease. Front Chem. (2017) 5:4. doi: 10.3389/fchem.2017.00004 28184370 PMC5266679

[32] White R Sotillo J Ancarola ME Borup A Boysen AT Brindley PJ. Special considerations for studies of extracellular vesicles from parasitic helminths: A community-led roadmap to increase rigour and reproducibility. J Extracell Vesicles. (2023) 12:12298. doi: 10.1002/jev2.12298 36604533 PMC9816087

[33] Théry C Clayton A Amigorena S Raposo and G. Isolation and characterization of exosomes from cell culture supernatants. Curr Protoc Cell Biol. (2006) 30:3.22:3.22.1–3.22.29. doi: 10.1002/0471143030.cb0322s30 18228490

[34] Iglesias L Valero A Benitez R Adroher FJ. *In vitro* cultivation of *Anisakis simplex:* pepsin increases survival and moulting from fourth larval to adult stage. Parasitology. (2001) 123:285–91. doi: 10.1017/S0031182001008423 11578092

[35] Polak I Stryiński R Podolska M Pawlak J Bittner MW Wiśniewski G. Drug efficacy on zoonotic nematodes of the Anisakidae family - new metabolic data. Parasitology. (2022) 149(8):1–45. doi: 10.1017/S0031182022000543 PMC10090616 35443901

[36] Mierzejewski K Kurzyńska A Golubska M Całka J Gałęcka I Szabelski M. New insights into the potential effects of PET microplastics on organisms via extracellular vesicle-mediated communication. Sci Total Environ. (2023), 904:166967. doi: 10.1016/j.scitotenv.2023.166967 37699490

[37] Webber J Clayton A. How pure are your vesicles? J Extracell Vesicles. (2013) 2:19861. doi: 10.3402/jev.v2i0.19861 PMC3760653 24009896

[38] Fiedorowicz E Markiewicz LH Sidor K Świątecka D Cieślińska A Matysiewicz M. The influence of breast milk and infant formulae hydrolysates on bacterial adhesion and Caco-2 cells functioning. Food Res Int. (2016) 89:679–88. doi: 10.1016/j.foodres.2016.09.022 28460966

[39] Stryiński R Mateos J Pascual S González ÁF Gallardo JM Łopieńska-Biernat E. Proteome profiling of L3 and L4 Anisakis simplex development stages by TMT-based quantitative proteomics. J Proteomics. (2019) 201:1–11. doi: 10.1016/j.jprot.2019.04.006 30978463

[40] Stryiński R Mateos J Łopieńska-Biernat E Carrera M. Shotgun Proteomics for L3 and L4 Anisakis simplex Development Stages. In: Carrera M Mateos J, editors. Shotgun Proteomics: Methods and Protocols, Methods in Molecular Biology. Springer, US (2021). p. 59–75. doi: 10.1007/978-1-0716-1178-4_5 33687709

[41] Niu M Cho J-H Kodali K Pagala V High AA Wang H. Extensive peptide fractionation and y 1 ion-based interference detection method for enabling accurate quantification by isobaric labeling and mass spectrometry. Anal Chem. (2017) 89:2956–63. doi: 10.1021/acs.analchem.6b04415 PMC5467445 28194965

[42] Mierzejewski K Stryiński R Łopieńska-Biernat E Mateos J Bogacka I Carrera M. A Complex Proteomic Response of the Parasitic Nematode Anisakis simplex s.s. to Escherichia coli Lipopolysaccharide. Mol Cell Proteomics. (2021) 20:100166. doi: 10.1016/j.mcpro.2021.100166 34673282 PMC8605257

[43] Stryiński R Mateos J Carrera M Jastrzębski JP Bogacka I Łopieńska-Biernat E. Tandem Mass Tagging (TMT) Reveals Tissue-Specific Proteome of L4 Larvae of Anisakis simplex s. s.: Enzymes of Energy and/or Carbohydrate Metabolism as Potential Drug Targets in Anisakiasis. Int J Mol Sci. (2022) 23:4336. doi: 10.3390/ijms23084336 35457153 PMC9027741

[44] Käll L Canterbury JD Weston J Noble WS MacCoss MJ. Semi-supervised learning for peptide identification from shotgun proteomics datasets. Nat Methods. (2007) 4:923–5. doi: 10.1038/nmeth1113 17952086

[45] Yu G Wang L-G Han Y He Q-Y. clusterProfiler: an R package for comparing biological themes among gene clusters. OMICS. (2012) 16:284–7. doi: 10.1089/omi.2011.0118 PMC3339379 22455463

[46] Luo W Brouwer C. Pathview: an R/Bioconductor package for pathway-based data integration and visualization. Bioinformatics. (2013) 29:1830–1. doi: 10.1093/bioinformatics/btt285 PMC3702256 23740750

[47] Doncheva NT Morris JH Gorodkin J Jensen LJ. Cytoscape stringApp: network analysis and visualization of proteomics data. J Proteome Res. (2019) 18:623–32. doi: 10.1021/acs.jproteome.8b00702 PMC6800166 30450911

[48] Maizels RM Smits HH McSorley HJ. Modulation of host immunity by helminths: the expanding repertoire of parasite effector molecules. Immunity. (2018) 49:801–18. doi: 10.1016/j.immuni.2018.10.016 PMC6269126 30462997

[49] Kochanowski M Dąbrowska J Różycki M Sroka J Karamon J Bełcik A. Proteomic profiling and in silico characterization of the secretome of anisakis simplex sensu stricto L3 larvae. Pathogens. (2022) 11:246. doi: 10.3390/pathogens11020246 35215189 PMC8879239

[50] Boysen AT Whitehead B Stensballe A Carnerup A Nylander T Nejsum P. Fluorescent labeling of helminth extracellular vesicles using an *in vivo* whole organism approach. Biomedicines. (2020) 8:213. doi: 10.3390/biomedicines8070213 32674418 PMC7399896

[51] Drurey C Maizels RM. Helminth extracellular vesicles: Interactions with the host immune system. Mol Immunol. (2021) 137:124–33. doi: 10.1016/j.molimm.2021.06.017 PMC8636279 34246032

[52] Harischandra H Yuan W Loghry HJ Zamanian M Kimber MJ. Profiling extracellular vesicle release by the filarial nematode Brugia malayi reveals sex-specific differences in cargo and a sensitivity to ivermectin. PloS Negl Trop Dis. (2018) 12:e0006438. doi: 10.1371/journal.pntd.0006438 29659599 PMC5919703

[53] Khosravi M Mirsamadi ES Mirjalali H Zali MR. Isolation and functions of extracellular vesicles derived from parasites: the promise of a new era in immunotherapy, vaccination, and diagnosis. Int J Nanomedicine. (2020) 15:2957–69. doi: 10.2147/IJN.S250993 PMC7196212 32425527

[54] Buck AH Blaxter M. Functional diversification of Argonautes in nematodes: an expanding universe. Biochem Soc Trans. (2013) 41:881–6. doi: 10.1042/BST20130086 PMC3782831 23863149

[55] Chow FW-N Koutsovoulos G Ovando-Vázquez C Neophytou K Bermúdez-Barrientos JR Laetsch DR. Secretion of an Argonaute protein by a parasitic nematode and the evolution of its siRNA guides. Nucleic Acids Res. (2019) 47:3594–606. doi: 10.1093/nar/gkz142 PMC6468290 30820541

[56] Zagoskin MV Wang J Neff AT Veronezi GMB Davis RE. Small RNA pathways in the nematode Ascaris in the absence of piRNAs. Nat Commun. (2022) 13:837. doi: 10.1038/s41467-022-28482-7 35149688 PMC8837657

[57] White R Kumar S Chow FW-N Robertson E Hayes KS Grencis RK. Extracellular vesicles from Heligmosomoides bakeri and Trichuris muris contain distinct microRNA families and small RNAs that could underpin different functions in the host. Int J Parasitol. (2020) 50:719–29. doi: 10.1016/j.ijpara.2020.06.002 PMC7435682 32659276

[58] Varshney D Petit A-P Bueren-Calabuig JA Jansen C Fletcher DA Peggie M. Molecular basis of RNA guanine-7 methyltransferase (RNMT) activation by RAM. Nucleic Acids Res. (2016) 44:10423–36. doi: 10.1093/nar/gkw637 PMC5137418 27422871

[59] Blazie SM Takayanagi-Kiya S McCulloch KA Jin Y. Eukaryotic initiation factor EIF-3.G augments mRNA translation efficiency to regulate neuronal activity. Elife. (2021) 10:e68336. doi: 10.7554/eLife.68336 34323215 PMC8354637

[60] Aziz RK Breitbart M Edwards RA. Transposases are the most abundant, most ubiquitous genes in nature. Nucleic Acids Res. (2010) 38:4207–17. doi: 10.1093/nar/gkq140 PMC2910039 20215432

[61] Palomba M Rughetti A Mignogna G Castrignanò T Rahimi H Masuelli L. Proteomic characterization of extracellular vesicles released by third stage larvae of the zoonotic parasite Anisakis pegreffii (Nematoda: Anisakidae). Front Cell Infect Microbiol. (2023) 13:1079991. doi: 10.3389/fcimb.2023.1079991 37009516 PMC10050594

[62] Hansen EP Fromm B Andersen SD Marcilla A Andersen KL Borup A. Exploration of extracellular vesicles from Ascaris suum provides evidence of parasite–host cross talk. J Extracell Vesicles. (2019) 8:1578116. doi: 10.1080/20013078.2019.1578116 30815237 PMC6383609

[63] Eichenberger RM Ryan S Jones L Buitrago G Polster R Montes de Oca M. Hookworm secreted extracellular vesicles interact with host cells and prevent inducible colitis in mice. Front Immunol. (2018) 9:850. doi: 10.3389/fimmu.2018.00850 29760697 PMC5936971

[64] Eichenberger RM Talukder MH Field MA Wangchuk P Giacomin P Loukas A. Characterization of Trichuris muris secreted proteins and extracellular vesicles provides new insights into host–parasite communication. J Extracell Vesicles. (2018) 7:1428004. doi: 10.1080/20013078.2018.1428004 29410780 PMC5795766

[65] Bautista D Rodríguez L-S Franco MA Angel J Barreto A. Caco-2 cells infected with rotavirus release extracellular vesicles that express markers of apoptotic bodies and exosomes. Cell Stress Chaperones. (2015) 20:697–708. doi: 10.1007/s12192-015-0597-9 25975376 PMC4463923

[66] Bellini I Scribano D Sarshar M Ambrosi C Pizzarelli A Palamara AT. Inflammatory response in caco-2 cells stimulated with anisakis messengers of pathogenicity. Pathogens. (2022) 11:1214. doi: 10.3390/pathogens11101214 36297271 PMC9611079

[67] Chaïbi C Cotte-Laffitte J Sandré C Esclatine A Servin AL Quéro A-M. Rotavirus induces apoptosis in fully differentiated human intestinal Caco-2 cells. Virology. (2005) 332:480–90. doi: 10.1016/j.virol.2004.11.039 15680413

[68] Dias C Ribeiro M Correia-Branco A Domínguez-Perles R Martel F Saavedra MJ. Virulence, attachment and invasion of Caco-2 cells by multidrug-resistant bacteria isolated from wild animals. Microb Pathog. (2019) 128:230–5. doi: 10.1016/j.micpath.2019.01.011 30615997

[69] Li C-W Su M-H Chen B-S. Investigation of the Cross-talk Mechanism in Caco-2 Cells during Clostridium difficile Infection through Genetic-and-Epigenetic Interspecies Networks: Big Data Mining and Genome-Wide Identification. Front Immunol. (2017) 8:901. doi: 10.3389/fimmu.2017.00901 28824629 PMC5539260

[70] Ma’ayeh SY Knörr L Sköld K Garnham A Ansell BRE Jex AR. Responses of the differentiated intestinal epithelial cell line caco-2 to infection with the giardia intestinalis GS isolate. Front Cell Infect Microbiol. (2018) 8:244. doi: 10.3389/fcimb.2018.00244 30062089 PMC6055019

[71] Ebner F Kuhring M Radonić A Midha A Renard BY Hartmann S. Silent witness: dual-species transcriptomics reveals epithelial immunological quiescence to helminth larval encounter and fostered larval development. Front Immunol. (2018) 9:1868. doi: 10.3389/fimmu.2018.01868 30158930 PMC6104121

[72] Carballeda-Sangiao N Sánchez-Alonso I Navas A Arcos SC de Palencia PF Careche M. Anisakis simplex products impair intestinal epithelial barrier function and occludin and zonula occludens-1 localisation in differentiated Caco-2 cells. PloS Negl Trop Dis. (2020) 14:e0008462. doi: 10.1371/journal.pntd.0008462 32628665 PMC7365482

[73] Napoletano C Mattiucci S Colantoni A Battisti F Zizzari IG Rahimi H. *Anisakis pegreffii* impacts differentiation and function of human dendritic cells. Parasite Immunol. (2018) 40:e12527. doi: 10.1111/pim.12527 29569735

[74] Messina CM Pizzo F Santulli A Bušelić I Boban M Orhanović S. Anisakis pegreffii (Nematoda: Anisakidae) products modulate oxidative stress and apoptosis-related biomarkers in human cell lines. Parasit Vectors. (2016) 9:607. doi: 10.1186/s13071-016-1895-5 27887635 PMC5124272

[75] Zamanian M Fraser LM Agbedanu PN Harischandra H Moorhead AR Day TA. Release of small RNA-containing exosome-like vesicles from the human filarial parasite brugia malayi. PloS Negl Trop Dis. (2015) 9:e0004069. doi: 10.1371/journal.pntd.0004069 26401956 PMC4581865

[76] Coakley G McCaskill JL Borger JG Simbari F Robertson E Millar M. Extracellular vesicles from a helminth parasite suppress macrophage activation and constitute an effective vaccine for protective immunity. Cell Rep. (2017) 19:1545–57. doi: 10.1016/j.celrep.2017.05.001 PMC5457486 28538175

[77] You H Stephenson RJ Gobert GN McManus DP. Revisiting glucose uptake and metabolism in schistosomes: new molecular insights for improved schistosomiasis therapies. Front Genet. (2014) 5:176. doi: 10.3389/fgene.2014.00176 24966871 PMC4052099

[78] You Y Zhou C Li D Cao Z-L Shen W Li W-Z. Sorting nexin 10 acting as a novel regulator of macrophage polarization mediates inflammatory response in experimental mouse colitis. Sci Rep. (2016) 6:20630. doi: 10.1038/srep20630 26856241 PMC4746623

[79] Dudás EF Huynen MA Lesk AM Pastore A. Invisible leashes: The tethering VAPs from infectious diseases to neurodegeneration. J Biol Chem. (2021) 296:100421. doi: 10.1016/j.jbc.2021.100421 33609524 PMC8005810

[80] Hermans D van Beers L Broux B. Nectin family ligands trigger immune effector functions in health and autoimmunity. Biol (Basel). (2023) 12:452. doi: 10.3390/biology12030452 PMC10045777 36979144

[81] Su C Cao Y Kaplan J Zhang M Li W Conroy M. Duodenal helminth infection alters barrier function of the colonic epithelium *via* adaptive immune activation. Infect Immun. (2011) 79:2285–94. doi: 10.1128/IAI.01123-10 PMC3125859 21444669

[82] Ballet R Emre Y Jemelin S Charmoy M Tacchini-Cottier F Imhof BA. Blocking Junctional Adhesion Molecule C Enhances Dendritic Cell Migration and Boosts the Immune Responses against Leishmania major. PloS Pathog. (2014) 10:e1004550. doi: 10.1371/journal.ppat.1004550 25474593 PMC4256467

[83] Issekutz TB Palecanda A Kadela-Stolarz U Marshall JS. Blockade of either alpha-4 or beta-7 integrins selectively inhibits intestinal mast cell hyperplasia and worm expulsion in response toNippostrongylus brasiliensis infection. Eur J Immunol. (2001) 31:860–8. doi: 10.1002/1521-4141(200103)31:3<860::AID-IMMU860>3.0.CO;2-9 11241291

[84] Liszewski MK Atkinson JP. Membrane cofactor protein (MCP; CD46): deficiency states and pathogen connections. Curr Opin Immunol. (2021) 72:126–34. doi: 10.1016/j.coi.2021.04.005 PMC8123722 34004375

[85] Nelson MM Jones AR Carmen JC Sinai AP Burchmore R Wastling JM. Modulation of the host cell proteome by the intracellular apicomplexan parasite. Toxoplasma gondii Infect Immun. (2008) 76:828–44. doi: 10.1128/IAI.01115-07 PMC2223483 17967855

[86] Sarkar A Pawar SV Chopra K Jain M. Gamut of glycolytic enzymes in vascular smooth muscle cell proliferation: Implications for vascular proliferative diseases. Biochim Biophys Acta (BBA) - Mol Basis Dis. (2024) 1870:167021. doi: 10.1016/j.bbadis.2024.167021 38216067

[87] Perez-Casal J Potter AA. Glyceradehyde-3-phosphate dehydrogenase as a suitable vaccine candidate for protection against bacterial and parasitic diseases. Vaccine. (2016) 34:1012–7. doi: 10.1016/j.vaccine.2015.11.072 26686572

[88] Morris SR Sakanari JA. Characterization of the serine protease and serine protease inhibitor from the tissue-penetrating nematode Anisakis simplex. J Biol Chem. (1994) 269:27650–6. doi: 10.1016/S0021-9258(18)47035-4 7961683

[89] Hotez PJ Pritchard DI. Hookworm infection. Sci Am. (1995) 272:68–74. doi: 10.1038/scientificamerican0695-68 7761817

[90] Malagón D Benítez R Kasšný M Adroher FJ. Peptidases in parasitic nematodes: a review. In: Erzinger GS, editor. Parasites: Ecology, Diseases and Management. Nova Science Publishers, Inc, NY, USA (2013). p. 61–102.

